# Transcriptome profiling of influenza A virus-infected lung epithelial (A549) cells with lariciresinol-4-β-D-glucopyranoside treatment

**DOI:** 10.1371/journal.pone.0173058

**Published:** 2017-03-08

**Authors:** Beixian Zhou, Jing Li, Xiaoli Liang, Zifeng Yang, Zhihong Jiang

**Affiliations:** 1 State Key Laboratory of Quality Research in Chinese Medicine, Macau Institute for Applied Research in Medicine and Health, Macau University of Science and Technology, Taipa, Macau, China; 2 State Key Laboratory of Respiratory Diseases, Guangzhou Institute of Respiratory Disease, National Clinical Centre of Respiratory Disease, The First Affiliated Hospital, Guangzhou Medical University, Guangzhou, People’s Republic of China; University of Hong Kong, HONG KONG

## Abstract

The influenza A virus is an acute contagious pathogen that affects the human respiratory system and can cause severe lung disease and even death. Lariciresinol-4-β-D-glucopyranoside is a lignan that is extracted from *Isatis indigotica*, which is a medicinal herb plant that was commonly applied to treat infections, the common cold, fever and inflammatory diseases. Our previous study demonstrated that lariciresinol-4-β-D-glucopyranoside possesses anti-viral and anti-inflammatory properties. However, the comprehensive and detailed mechanisms that underlie the effect of lariciresinol-4-β-D-glucopyranoside interventions against influenza virus infection remain to be elucidated. In this study, we employed high-throughput RNA sequencing (RNA-seq) to investigate the transcriptomic responses of influenza A virus-infected lung epithelial (A549) cells with lariciresinol-4-β-D-glucopyranoside treatment. The transcriptome data show that infection with influenza A virus prompted the activation of 368 genes involved in RIG-I signalling, the inflammatory response, interferon α/β signalling and gene expression that was not affected by lariciresinol-4-β-D-glucopyranoside treatment. Lariciresinol-4-β-D-glucopyranoside exerted its pharmacological actions on the immune system, signal transduction, cell cycle and metabolism, which may be an underlying defense mechanism against influenza virus infection. In addition, 166 differentially expressed genes (DEGs) were uniquely expressed in lariciresinol-4-β-D-glucopyranoside-treated cells, which were concentrated in the cell cycle, DNA repair, chromatin organization, gene expression and biosynthesis domains. Among them, six telomere-associated genes were up-regulated by lariciresinol-4-β-D-glucopyranoside treatment, which have been implicated in telomere regulation and stability. Collectively, we employed RNA-seq analysis to provide comprehensive insight into the mechanism of lariciresinol-4-β-D-glucopyranoside against influenza virus infection.

## Introduction

The ongoing battle against infectious diseases such as influenza is an enduring process. Type A influenza virus causes epidemics each year and has contributed to hundreds of thousands of deaths worldwide. Due to the viruses’ gene mutations and genome reassortments, the viruses’ variants have easily acquired resistance to current antiviral medications [1, 2]. Several studies have documented the recent emergence of neuraminidase (NA) inhibitor-resistant avian-origin influenza A (H7N9) strains [3, 4].

Influenza A virus infection in the lung is associated with a robust host inflammatory response that is characterized by abundant leukocyte infiltration (e.g., T cells, macrophages, and monocytes) and sustained elevated levels of inflammatory immune mediators, including tumour necrosis factor-α (TNF-α), IL-6, and interferon gamma-induced protein 10 (IP-10) [5–7]. Low or high virulent influenza virus infection alters the expression of thousands of host cell genes, such as genes related to cell cycle progression, apoptosis, inflammatory response, and metabolism [8–11]. In general, the complex interactions between the virulence factors of the pathogen and the host immune defence were associated with the infectious disease outcome [12]. Therefore, the analysis of the global changes in the host gene expression profile during influenza A virus infection may contribute to the identification of potential novel therapeutic targets that are urgently required for preventing influenza-associated diseases.

Chinese herbal medicine (CHM) has served a critical role in preventing and controlling influenza infection disease in China since ancient times [13]. Current medical plants contain numerous components that serve as a source for the development of antiviral agents. Basic research of medicinal herbs primarily focuses on bioactive compound discovery and the utilization of cell culture or animal models of influenza infection to study their pharmacological mechanisms of action. The investigation of how these bioactive compounds exert their therapeutic effect and restore body system homeostasis is substantially based on the investigators’ deductive assumptions, which may result in numerous biases and inaccuracies. These unsystematic and biased findings from research may pose drug safety concerns and even harm to patients’ health in the future. For instance, shikimic acid from Illicium verum is a primary precursor for the synthesis of oseltamivir; however, oseltamivir has been reported to cause neuropsychiatric side effects [14]. Yang et al. has suggested the application of modern technologies, such as transcriptomic technologies, to study the bioactive compounds from traditional Chinese medicine (TCM) [15].

RNA-sequencing (RNA-seq), which is a genome-wide analytical technology, has been extensively utilized to analyse the transcriptome of various infectious diseases, including influenza A virus infection [16, 17]. RNA-seq analysis of the host response to human or avian influenza virus infection has been performed in the mouse, crow, chicken and swine and has provided insights into influenza disease pathogenesis [18–21]. RNA-seq offers a systematic approach for delineating gene expression changes at a given point in time [22], which may identify the key molecular events that are linked to the pathogenesis of influenza virus infection and provide a comprehensive analysis of the pharmacological effects of herbal components.

The herb *Isatis indigotica* (Ban-Lan-Gen) is a common Chinese herb that is used as an important and popular herbal remedy for the clinical treatment of the common cold, fever and influenza [23]. A number of active compounds have been identified in *I*. *indigotica*, including indole alkaloids, flavonoids, and lignans [24, 25]. Our previous studies have demonstrated that a lignan from *I*. *indigotica*, lariciresinol-4-β-D-glucopyranoside, exhibited potent inhibitory activity against influenza virus A/PR/8/34(H1N1), with 50% inhibitory concentrations (IC_50_) of 50 μg/mL and selectively inedx (SI) more than 4, while it showed low cytotoxic potency towards MDCK cells for which the CC_50_ exceeded 200 μg/mL [26]. Furthermore, lariciresinol-4-β-D-glucopyranoside suppressed influenza A (H1N1) virus-infected or TNF-α-stimulated activation of NF-κB in a NF-κB luciferase reporter stable HEK293 cell line. Meanwhile, the increased level of pro-inflammatory genes in influenza A H1N1 or H9N2 viruses-infected cells, such as TNF-α, IL-6, IP-10 and IL-8, were inhibted by lariciresinol-4-β-D-glucopyranoside treatment [26]. Therefore, our previous results suggested that lariciresinol-4-β-D-glucopyranoside impaired influenza virus propagation and suppressed the human or avian influenza virus-induced host inflammatory response via the regulation of NF-κB activation [26]. In this study, we used RNA-seq technology to systematically assess the transcriptome profile of influenza A virus-infected lung epithelial (A549) cells following lariciresinol-4-β-D-glucopyranoside treatment, which may result in gaining a comprehensive understanding the mechanism of lariciresinol-4-β-D-glucopyranoside against influenza A virus infection.

## Materials and methods

### Compounds, cell culture, virus infection and sample preparation

The compound lariciresinol-4-β-D-glucopyranoside was prepared as previously described, dissolved in dimethyl sulfoxide (DMSO) (Sigma) as a stock solution of 50 mg/ml and stored at -20°C until use. A549 human lung adenocarcinoma cells that were purchased from ATCC were cultured in MDEM/DF12 (1:1, V/V), with 10% foetal bovine serum (FBS), 100 U/ml penicillin, and 100 μg/ml streptomycin under standard conditions at 37°C in 5% CO2 humidified air. The A549 cells were grown in a monolayer to 80% to 90% confluency and detached from the flask using 10 mM EDTA (pH 7.4) and 0.25% trypsin. The cells were harvested, and 5 × 10^5^ A549 cells were seeded in 6-well tissue culture plates. The following day, the cells were washed twice with PBS and infected with A/PR/8/34 (H1N1) at multiplicities of infection (MOIs) of 0.1 using serum-free medium for 2 hours at 37°C. The inoculum was removed, and the cells were treated with or without lariciresinol-4-β-D-glucopyranoside (400 μg/ml to 800 μg/ml). After 24 h, the cells were lysed in TRIzol reagent (Life technologies) and stored at -80°C.

### RNA isolation, cDNA library construction and sequencing

Total RNA extracts from each sample were obtained following the manufacturer’s instructions (Life technologies). The total RNA quality was assessed by 1.5% agarose gel electrophoresis. The A260/A280 ratio was determined using a N0061noDrop ND-200 spectrophotometer (NanoDrop Technologies, USA). RNA integrity was assessed by Agilent 2200 TapeStation analysis (Agilent Technologies, Santa Clara, CA, USA). A A260/A280 ratio between 1.8 to 2.0 and RIN > 7 were considered acceptable. RNA sequencing was performed on an Illumina HiSeq-2000 RNA-seq sequence production system. RNA-seq data of this study have been submitted to the NCBI Gene Expression Omnibus with the accession number GSE93999 (https://www.ncbi.nlm.nih.gov/geo/).

### Pathway analysis of differentially expressed genes

We performed Gene Ontology (GO) and pathway enrichment analysis using the Database for Annotation, Visualization, and Integrated Discovery (DAVID) to obtain a list of differentially expressed genes. We identified GO terms and the Kyoto Encyclopaedia of Genes and Genomes (KEGG) pathways that were significantly enriched on our list of altered genes (P < 0.05). Additionally, the integrated pathways with statistical values were computed from our list of DEGs using a reactome pathway analysis (http://www.reactome.org/).

## Results

### Sequencing and alignment

**Fig 1:**
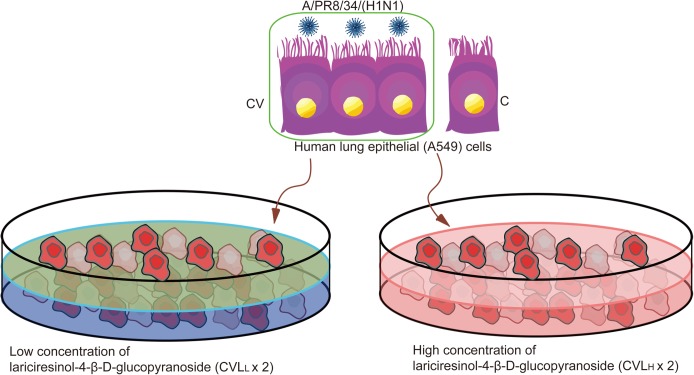
Experimental design and RNA-seq. The study groups were as follows: one group lung epithelial (A549) cells without infection (C), one group of A549 cells infected with A/PR8/34/(H1N1) (CV), two groups of A549 cells infected with A/PR8/34/(H1N1) at the low concentration of lariciresinol-4-β-D-glucopyranoside treatment (400 μg/ml) (CVL_L_), and two groups of A549 cells infected with A/PR8/34/(H1N1) at the high concentration of lariciresinol-4-β-D-glucopyranoside treatment (800 μg/ml) (CVL_H_).

To understand the molecular mechanism of lariciresinol-4-β-D-glucopyranoside action against influenza A virus infection, we performed RNA-seq of influenza A virus-infected human lung epithelial (A549) cells in the absence or presence of different concentrations of lariciresinol-4-β-D-glucopyranoside treatment using an Illumina HiSeq 2000 platform, and then, the sequences were aligned against human gene sequences (hg19, **Table 1**). There were four experimental conditions, including lung epithelial (A549) cells without infection (C), A549 cells infected with A/PR8/34/(H1N1) (CV), A549 cells infected with A/PR8/34/(H1N1) at the low concentration of lariciresinol-4-β-D-glucopyranoside treatment (400 μg/ml) (CVL_L_ × 2), and A549 cells infected with A/PR8/34/(H1N1) at a high concentration of lariciresinol-4-β-D-glucopyranoside treatment (800 μg/ml) (CVL_H_ × 2) (**Fig 1**). Then, 24 hours after influenza virus infection, samples were harvested and subjected to library construction and sequencing. More than 13 million raw reads for each sample were generated from constructed RNA-seq libraries using the Illumina HiSeq 2000 platform. After stringent data filtering, 12.29 M (93.04%, C), 12.94 M (92.17%, CV), 12.35 M (91.50%, CVL_L_) and 12.56 M (90.71%, CVL_H_) sequencing reads can be unambiguously mapped against the reference genome (**Table 1**), which suggests the high quality of the sequencing of these samples and a satisfactory variable for additional analysis.

**Table 1:** Statistics of RNA-seq data and mapped reads obtained by an RNA-Seq analysis of influenza A virus (H1N1)-infected lung epithelial (A549) cells with or without lariciresinol-4-β-D-glucopyranoside treatment.

Sample type	C	CV	CVL_L_	CVL_H_	CVL_L_	CVL_H_
Total reads	13,216,449 (100%)	14,035,807 (100%)	13,497,899 (100%)	13,841,371 (100%)	13,551,998 (100%)	13,279,470 (100%)
Total mapped (%)	12,961,879 (98.07%)	13,652,450 (97.27%)	13,045,031 (96.64%)	13,274,612 (95.91%)	13,034,539 (96.18%)	12,914,700 (97.25%)
Multiple mapped (%)	665,860 (5.04%)	715,719 (5.10%)	694,903 (5.15%)	719,288 (5.20%)	723,168 (5.34%)	646,251 (4.87%)
Uniquely mapped (%)	12,296,019 (93.04%)	12,936,731 (92.17%)	12,350,128 (91.50%)	12,555,324 (90.71%)	12,311,371 (90.85%)	12,268,449 (92.39%)
Reads map to “+”	6,311,551 (47.76%)	6,657,196 (47.43%)	6,343,777 (47.00%)	6,455,222 (46.64%)	6,336,575 (46.76%)	6,298,126 (47.43%)
Reads map to “-”	6,650,328 (50. 32%)	6,995,254 (49.84%)	6,701,254 (49.65%)	6,819,390 (49.27%)	6,697,964 (49.42%)	6,616,574 (49.83%)

### Identification of differentially expressed genes (DEGs)

**Fig 2:**
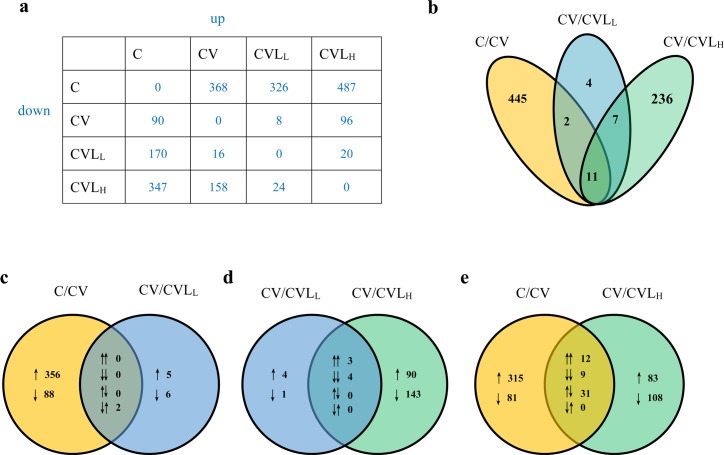
Global overview of the RNA-seq data of influenza A virus-infected lung epithelial (A549) cells with or without lariciresinol-4-β-D-glucopyranoside treatment. **(a)** Summary of the number of more than 1.5 fold up- or down-regulated differentially expressed genes (DEGs) as determined by RNA-Seq in all four experimental conditions. For example, 368 DEGs were up-regulated in CV/C, and 90 DEGs were down-regulated in CV/C. **(b)** The Venn diagram shows the total number of overlapping DEG profiles for three comparisons (C/CV, CV/CVL_L_, CV/CVL_H_). **(c-e)** Detailed analysis of up-regulated and down-regulated genes among the three comparisons, e.g., comparisons of C/CV and CV/CVL_H_, as shown in **Fig 1E**, the single up arrow (315↑) or single down arrow (81↓) denotes 315 up-regulated DEGs or 81 down-regulated DEGs, respectively. Downwards paired arrows (12↓↓) or upwards paired arrows (9↑↑) in an overlapping area denote 12 common up-regulated DEGs or nine common down-regulated DEGs between the comparisons. An upwards arrow leftwards of a downwards arrow (31↑↓) denotes 31 up-regulated DEGs in C/CV but down-regulated in CV/CVL_H_. Similarly, a downwards arrow leftwards of an upwards arrow (0↓↑) denotes no up-regulated DEGs in C/CV but down-regulated DEGs in CV/CVL_H_.

**Fig 3:**
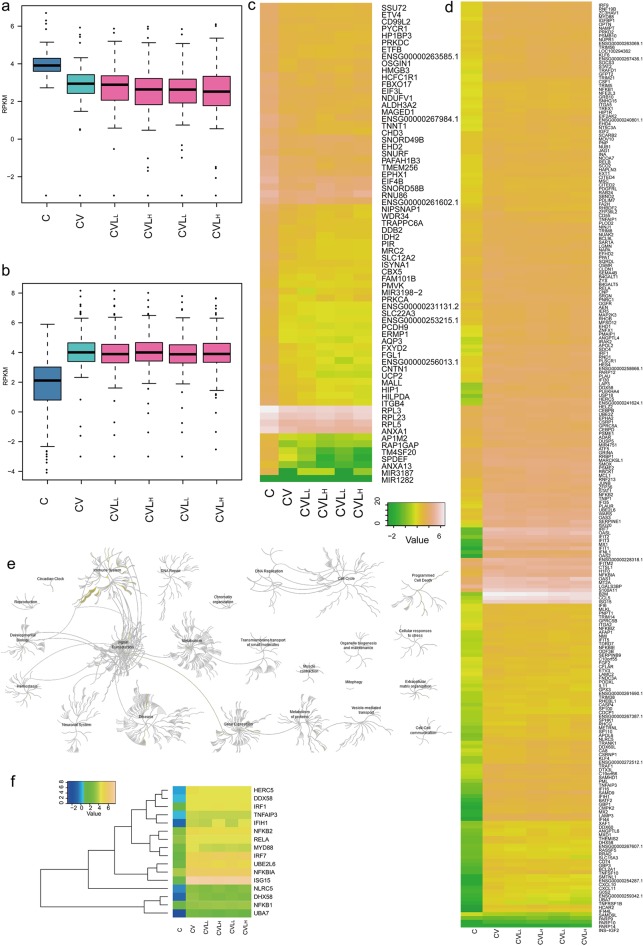
DEGs by host defence. **a) C**omparison of 125 down-regulated genes induced by influenza virus infection among five samples. **b) C**omparison of 243 up-regulated genes induced by influenza virus infection among five samples. **c)** Heatmap showing 125 down-regulated genes induced by influenza virus infection but not altered by lariciresinol-4-β-D-glucopyranoside treatment (high expression: yellow; low expression: green). **d)** Heatmap showing 243 up-regulated genes induced by influenza virus infection but not altered by lariciresinol-4-β-D-glucopyranoside treatment among five samples (high expression: yellow; low expression: green). **e)** Enrichment analysis results of 327 up-regulated genes induced by influenza virus infection but not altered by lariciresinol-4-β-D-glucopyranoside treatment using the reactome database. **f)** Heatmap showing 16 DEGs in the RIG-I-MDA5-mediated induction of IFN-alpha-beta pathway (high expression: yellow; low expression: green).

Human lung epithelial (A549) cells with influenza A virus infection resulted in the greatest degree of differential expression (CV/C). A total of 458 genes (368 genes up-regulated, 90 genes down-regulated) were detected to be significantly altered (≥ 1.5-fold change, P < 0.05) (**Fig 2A**). Additionally, 368 (243 up-regulated and 125 down-regulated) of 458 genes were identified in all lariciresinol-4-β-D-glucopyranoside treatments (**S1 Table** and **Fig 3A–3D**), which reveals that the expression of these genes was triggered by host-pathogen interaction genes during influenza virus infection but was not regulated by lariciresinol-4-β-D-glucopyranoside treatments. To obtain a detailed understanding of the DEGs regulated by lariciresinol-4-β-D-glucopyranoside, we compared all treatments (CVL_L_ and CVL_H_) to CV. We identified 645 significantly altered DEGs (487 genes up-regulated and 158 genes down-regulated, P < 0.05) in the CVL_H_/CV comparison; however, the CVL_L_/CV comparison indicated that relatively few genes were significantly changed (8 up-regulated and 16 down-regulated, P < 0.05) (**Fig 2A**), which suggests that lariciresinol-4-β-D-glucopyranoside treatment exerts its effect against influenza virus infection in a dose-dependent manner. In addition, 13 genes were commonly overlapped and regulated in both pairwise comparison C/CV and CV/CVL_L_ (**Fig 2B**). Similarly, a comparison of the pairwise comparisons C/CV and CV/CVL_H_, 11 overlapping DEGs strongly revealed that lariciresinol-4-β-D-glucopyranoside treatment against virus infection does not regulate the large components of host genes that drive expression by viruses (**Fig 2B**). Interestingly, 243 DEGs were solely affected by the high concentration of lariciresinol-4-β-D-glucopyranoside treatment (CV/CVL_H_). A detailed analysis of the up-regulated and down-regulated genes among the three comparisons was performed (**Fig 2C–2E**). Based on the high concentration of lariciresinol-4-β-D-glucopyranoside treatment, 83 genes and 108 genes in the C/CV and CV/CVL_H_ comparisons were up-regulated and down-regulated, respectively (**Fig 2E**). These findings indicate that the effects of lariciresinol-4-β-D-glucopyranoside against virus infection were not attributed to regulation of the genes induced by viruses.

### DEGs in lung epithelial (A549) cells with influenza A virus infection

As previously mentioned, 369 DEGs were triggered by influenza virus infection but were not affected by lariciresinol-4-β-D-glucopyranoside treatment (**Fig 3A–3D** and **S1 Table**). To better understand the host cell response to influenza virus infection at 24 hours, we characterized the 368 DEGs data by performing the enrichment analysis using the DAVID and reactome pathway database. GO enrichment analysis suggested that these DEGs were related to the immune system, such as the response to wounding (GO:0009611), defence responses (GO:0006952), inflammatory response (GO:0006954), response to virus (GO:0009615) and immune response (GO:0006955) (**Fig 3E** and **S2 Table**).

Results from the enrichment analysis of the reactome indicated that 243 up-regulated genes were also significantly enriched in 60 pathways related to the immune system, disease, cell cycle and programmed cell death (P < 0.01, FDR < 0.05), such as antiviral defense, innate immunity, type I interferon signaling pathway and cytokine signaling in immune system (**Fig 3E** and **S2 Table**). There were 16 DEGs that were enriched in the RIG-I-like receptor signalling pathway (**Fig 3F** and **S2 Table**), which activates signalling cascades to produce type I interferon (IFN-α/β) and mediate the anti-viral response. These DEGs, which mainly included HERC5, DDX58, IRF1, IFR7, and ISG15, were reported to be involved in initiating the antiviral programme. DDX58 (RIG-I) recognized intracellular virus-derived RNA molecules and activated host defence responses in RNA-virus-infected cells [27]. Activation of the RIG-I signalling pathway triggers the signalling pathway that leads to the activation of the transcription factors NF-κB and IRF3/7, which transcribe cytokines and chemokines such as IFN-β, IL-8, TNF-α, IFN-λ1/2 and RANTES [28]. Robust type I interferon production by virus infection requires the IRF7-RIG-I amplification loop and the stability of RIG-I via post-translational modification by type I IFN [29, 30]. Interferon signalling is essential for the production of anti-viral mediators (such as OAS1, Mx1, SP100, and TRIM22) to defend against virus infection [31, 32]. The IFN-stimulated gene TRIM22 exerts its antiviral activity via polyubiquitination and degradation of viral nucleoprotein (NP) [32], whereas the viruses have evolved numerous strategies to counteract the interferon-mediated anti-viral response [33].

125 down-regulated genes that are significantly enriched in 12 pathways are involved in metabolism and gene expression (P < 0.01, FDR < 0.05), such as L13a-mediated translational silencing of ceruloplasmin expression, eukaryotic translation initiation and cap-dependent translation initiation (**S1 Fig** and **S3 Table**). The generation of a capped RNA primer from the host cell pre-mRNAs by the endonuclease activity of PB2 polymerase subunit is required for initiating viral mRNA transcription and causes the degradation of cellular mRNA [34]. Influenza virus protein translation is completely dependent on the host cell protein synthesis machinery, whereas the host’s cellular protein synthesis is shut down during the infection [35].

### Pattern against viral infections by lariciresinol-4-β-D-glucopyranoside

We determined that the regulation of gene expression effective against viral infections by lariciresinol-4-β-D-glucopyranoside has been demonstrated in two different ways: (i) direct regulation of the DEGs induced by influenza A virus infection, and (ii) regulation of the expression of other genes that are not affected by viruses to exert its effects.

#### Influenza A virus-induced DEGs directly regulated by lariciresinol-4-β-D-glucopyranoside treatment

**Fig 4:**
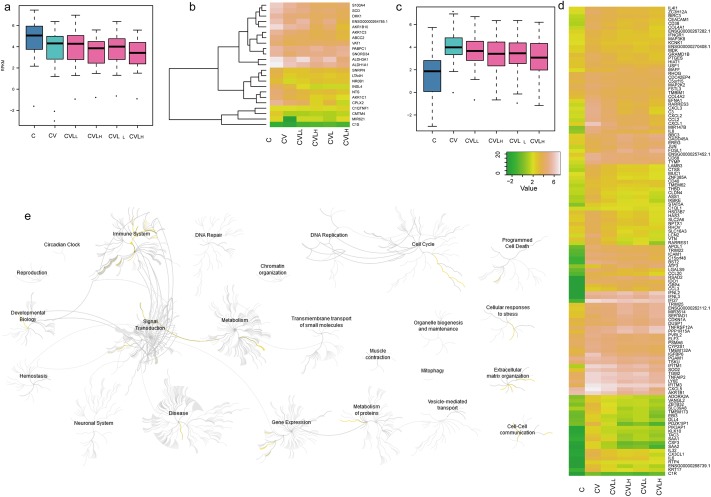
Bioinformatics analysis of the DEGs regulated by lariciresinol-4-β-D-glucopyranoside treatment. **a)** Comparison of 117 down-regulated genes regulated by lariciresinol-4-β-D-glucopyranoside treatment among five samples. **b)** Heatmap showing 23 DEGs regulated by lariciresinol-4-β-D-glucopyranoside treatment among five samples (high expression: yellow; low expression: green). **c)** Comparison of 29 up-regulated genes regulated by lariciresinol-4-β-D-glucopyranoside treatment among five samples. **d)** Heatmap showing 121 DEGs regulated by lariciresinol-4-β-D-glucopyranoside treatment among five samples (high expression: yellow; low expression: green). **e)** Enrichment analysis results of 146 DEGs regulated by lariciresinol-4-β-D-glucopyranoside treatment using the reactome database.

Using a criteria for data analysis (fold change ≥ ± 1.5 and p-value < 0.05), 146 DEGs (included 29 genes up-regulated and 117 genes down-regulated) have been identified significant difference between two comparison (CV/CVL_L_, CV/CVL_H_) (**Fig 4A–4D and S4 Table)**. We performed the enrichment analysis on the DEGs that were directly regulated by lariciresinol-4-β-D-glucopyranoside. The GO enrichment analysis revealed that the 146 DEGs were significantly enriched in the immune response, cell cycle, metablism, cellular responses to stress and cell-cell communication (**Fig 4E**), such as negative regulation of viral genome replication (GO:0045071), chemokine interleukin-8-like domain (IPR001811), GO terms related to chemotaxis (GO:0006935, GO:0060326 and GO:0008009) and defense response to virus (GO:0051607) **(S5 Table)**. We discovered that the expression of genes involved in the inflammatory response was down-regulated after lariciresinol-4-β-D-glucopyranoside treatment, such as CXCL1, CXCL2, CXCL3, CXCL5, IL-8, CCL2 and CX3CL1. Interestingly, GBP4 gene expression (gene name, guanylate binding protein 4; fold change = 2.76; p value = 3.22E-05) was up-regulated after treatment with lariciresinol-4-β-D-glucopyranoside (**Fig 4D** and **S4 Table)**. GBPs are a group of IFN-induced GTPases that are essential for the host antiviral defence. GBP1 has been extensively investigated and has been shown to act against many viruses, including vesicular stomatitis virus (VSV), encephalomyocarditis virus (ECMV) and hepatitis C virus (HCV) [36, 37]. In addition to proinflammatory cytokine genes, influenza virus infection also elicits the transcription of gene-encoding lipid mediators (including COX, LTA4H and PGES) [38–40]. The lariciresinol-4-β-D-glucopyranoside treatment down-regulated influenza virus-induced expression of PTGES (gene name, prostaglandin E-synthase; fold change = 3.33; p value = 1.73E-06). Increases in PTGES expression cause the generation of its metabolic product, prostaglandin E2 (PGE2), which has been shown to be involved in increasing viral replication and inhibiting type I IFN secretion [38]. KEGG pathway enrichment analysis showed that the down-regulated genes are enriched in the chemokine signaling pathway, metabolic pathways and toll-like receptor signaling pathway **(S5 Table** and **S6 Table)**, such as interleukin-7 signalling, prolactin receptor signalling and NOTCH2 activation and the transmission of signals to the nucleus.

#### Emerging DEGs by lariciresinol-4-β-D-glucopyranoside treatment

**Fig 5:**
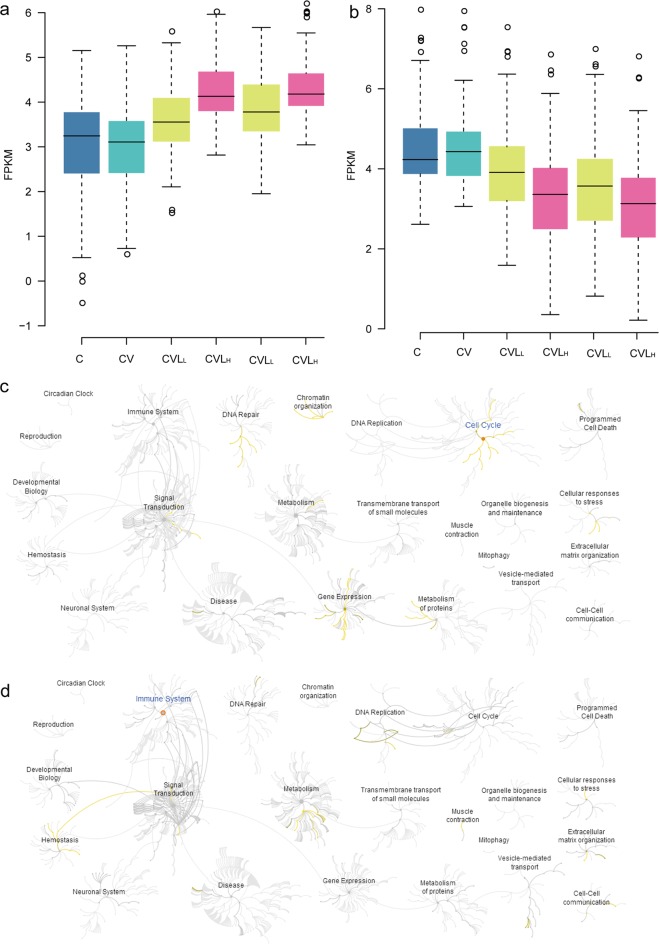
Bioinformatics analysis of the emerging DEGs that regulated by lariciresinol-4-β-D-glucopyranoside treatment. **a)** Comparison of 70 up-regulated genes that regulated by lariciresinol-4-β-D-glucopyranoside treatment among five samples but no significant change by influenza virus infection. **b)** Comparison of 70 down-regulated genes that regulated by lariciresinol-4-β-D-glucopyranoside treatment among five samples but no significant change by influenza virus infection. **c)** Enrichment analysis results of 70 up-regulated genes regulated by lariciresinol-4-β-D-glucopyranoside treatment using the reactome database. **d)** Enrichment analysis results of 96 down-regulated genes regulated by lariciresinol-4-β-D-glucopyranoside treatment using the reactome database.

Genes expression that was up-regulated or down-regulated at least 1.5-fold (fold change ≥ ± 1.5, p-value less than 0.05), but not in accordance with this criteria in virus-infected group (CV), which were defined as emerging DEGs by lariciresinol-4-β-D-glucopyranoside treatment. A total of 166 DEGs (70 up-regulated genes and 96 down-regulated genes) belong to the emerging DEGs that are considered to be indirectly effective genes that are not induced by viruses (**Fig 5A and 5B** and **S7 Table**). For the up-regulated genes, they are significantly enriched in the response to extracellular stimuli (GO:000999) and the response to nutrient (GO:0031667, GO:0007584) in the DAVID results (**S8 Table**). Pathway analysis using KEGG revealed that these DEGs were primarily concentrated in the cell cycle, DNA repair, chromatin organization and gene expression (**Fig 5C**) including 56 pathways (P<0.01, FDR < 0.05), such as the packaging of telomere ends, HDACs deacetylation of histones and RNA polymerase I promoter opening (**Table 2**).

**Table 2:** Enriched KEGG pathway of DEGs in lariciresinol-4-β-D-glucopyranoside treatment.

Pathway name	Entities found	Entities total	Entities P-Value	Entities FDR
Packaging of telomere ends	10	32	3.14E-13	5.79E-11
HDACs deacetylate histones	12	63	3.83E-13	5.79E-11
RNA polymerase I promoter opening	9	32	1.27E-11	1.06E-09
HATs acetylate histones	13	110	1.41E-11	1.06E-09
DNA methylation	9	35	2.79E-11	1.47E-09
DNA damage/telomere stress-induced senescence	11	70	2.94E-11	1.47E-09
Meiotic synapsis	10	60	1.39E-10	5.97E-09
PRC2 methylates histones and DNA	9	43	1.68E-10	6.23E-09
SIRT1 negatively regulates rRNA expression	9	44	2.06E-10	6.79E-09
Formation of the beta-catenin: TCF transactivating complex	10	64	2.58E-10	7.73E-09
Activated PKN1 stimulates transcription of AR (androgen receptor) regulated genes KLK2 and KLK3	9	47	3.65E-10	9.84E-09
RMTs methylate histone arginines	9	49	5.23E-10	1.31E-08
Telomere maintenance	10	73	9.02E-10	1.86E-08
Condensation of prophase chromosomes	9	53	1.03E-09	1.86E-08
Nucleosome assembly	9	53	1.03E-09	1.86E-08
Deposition of new CENPA-containing nucleosomes at the centromere	9	53	1.03E-09	1.86E-08
Transcriptional regulation by small RNAs	10	77	1.50E-09	2.54E-08
Meiotic recombination	9	58	2.24E-09	3.59E-08
RNA polymerase I chain elongation	9	59	2.60E-09	3.89E-08
meiosis	10	91	7.21E-09	1.08E-07
Chromosome maintenance	10	101	1.91E-08	2.55E-07
Chromatin modifying enzymes	14	241	1.96E-08	2.55E-07
Chromatin organization	14	241	1.96E-08	2.55E-07
RHO GTPases activate PKNs	9	77	2.52E-08	3.02E-07
NoRC negatively regulates rRNA expression	9	79	3.13E-08	3.75E-07
RNA polymerase I promoter clearance	9	81	3.86E-08	4.25E-07
Gene silencing by RNA	10	111	4.58E-08	5.04E-07
RNA polymerase I transcription	9	84	5.25E-08	5.25E-07
Senescence-associated secretory phenotype (SASP)	9	85	5.79E-08	5.79E-07
Negative epigenetic regulation of rRNA expression	9	88	7.75E-08	7.75E-07
Oxidative stress-induced senescence	9	107	3.95E-07	3.55E-06
Epigenetic regulation of gene expression	9	114	6.65E-07	5.99E-06
Cellular senescence	11	190	7.54E-07	6.79E-06
PERK regulates gene expression	6	38	1.07E-06	8.57E-06
RNA polymerase I, RNA polymerase III, and mitochondrial transcription	9	121	1.08E-06	8.67E-06
Mitotic prophase	9	128	1.71E-06	1.37E-05
ATF4 activates genes	5	32	9.26E-06	7.41E-05
TCF-dependent signalling in response to WNT	10	212	1.47E-05	1.03E-04
Cellular responses to stress	13	455	1.35E-04	9.45E-04
Nonhomologous end-joining (NHEJ)	5	57	1.40E-04	9.83E-04
G2/M DNA damage checkpoint	5	57	1.40E-04	9.83E-04
Recruitment and ATM-mediated phosphorylation of repair and signalling proteins at DNA double strand breaks	5	65	2.57E-04	0.001796963
ATF6-alpha activates chaperone genes	3	15	3.11E-04	0.002176276
DNA double-strand break response	5	73	4.35E-04	0.002608677
ATF6-alpha activates chaperones	3	17	4.47E-04	0.002682118
Signalling by Wnt	10	326	4.90E-04	0.00294052
RHO GTPase effectors	9	295	9.84E-04	0.005904968
Processing of DNA double-strand break ends	5	90	0.001106	0.006633812
G2/M checkpoints	5	92	0.001218	0.007306391
M Phase	9	306	0.001268	0.007606081
Unfolded protein response (UPR)	6	149	0.001829	0.009145478
Amino acid synthesis and interconversion (transamination)	4	61	0.001947	0.009737152
HDR through homologous recombination (HR) or single strand annealing (SSA)	5	122	0.004091	0.020454387
Homology directed repair	5	128	0.004998	0.02499001
Signalling by Rho GTPases	9	408	0.008327	0.041636644
Serine biosynthesis	2	16	0.008486	0.042427933

Six DEGs were located in the packaging of telomere ends pathway, including HIST1H2AC, HIST1H2BC, HIST1H2BJ, MID1IP1 and TERF2IP (**S5 Fig**). Histones (HIST1H2AC, HIST1H2BC, and HIST1H2BJ) have been identified as essential for transcription regulation, DNA repair, DNA replication and chromosomal stability. Telomeres serve a critical role in chromosome end-protection and genomic stability. Telomeric Repeat Binding Factor 2, Interacting Protein (TERF2IP) functions as a regulator of telomeres and transcription and is involved in the regulation of telomere length and protection [41]. TERF2IP is essential for negatively regulating telomere recombination and repressing homology-directed repair (HDR), which can affect telomere length [41].

For the down-regulated genes, they were primarily enriched in the biosynthetic processes (**S8 Table**), such as lipid (GO:0008610), cholesterol, steroid and sterol biosynthesis in the DAVID results. In the reactome results, they were primarily enriched in the domains of metabolism, haemostasis and DNA replication (**Fig 5D**) including 45 pathways, such as the regulation of cholesterol biosynthesis by SREBP (SREBF) and cholesterol biosynthesis (**S9 Table**). The key G1-phase cell-cycle regulator CCND3 (gene name, cyclin D3; fold change = 4.59; p value = 1.61E-16) was down-regulated in lariciresinol-4-β-D-glucopyranoside-treated cells.

## Discussion

Traditional Chinese medicine (TCM) may be an alternative approach for the treatment of influenza virus infection. Although the underlying mechanism remains elusive, substantial clinical evidence that clearly demonstrates the beneficial effect of TCM on influenza diseases is available. Due to the complex interactions network between TCM compounds and their anti-influenza targets, the transcriptome sequencing (RNA-seq) technology offers a systematic analysis for the study of TCM pharmacology. To gain insight into the molecular mechanism of lariciresinol-4-β-D-glucopyranoside action against influenza virus infection, our present study for the first time employed RNA-seq technology to analyse the global gene expression profiling of human alveolar epithelial (A549) cells that relate to the host antiviral response.

Host gene expression profiling analysis revealed association of activated inflammatory response and defence responses in influenza A virus-infected cells (**S2 Table** and **Fig 3E**). Moreover, our results also proved that the expression of RIG-I signalling or antiviral response-related genes, such as DDX58, NLRC5, IRF7, ISG15 and TNFAIP3 elevated significantly at 24 h after influenza virus infection (**Fig 3F** and **S2 Table**). All these data are consistent with previous findings that genes involved in innate immunity and pro-inflammatory responses were elicited by challenge with human or avian influenza A virus [20, 42, 43].

Go enrichment analysis showed that the DEGs directly regulated by lariciresinol-4-β-D-glucopyranoside treatment were mainly located in immune system and defense response to virus (GO:0051607) (**Fig 4E** and **S5 Table**). We observed that influenza A virus infection induced up-regulated expression of cytokines/chemokines, such as IL-6, IL8, CCL2, CX3CL1, CCL3 and CXCL3, were all decreased upon lariciresinol-4-β-D-glucopyranoside treatment (**Fig 4B**, **Fig 4D** and **S4 Table**). IKBKE (also known as IKK-i), an IKK-related kinase, had a 4.94-fold decrease in lariciresinol-4-β-D-glucopyranoside treatment group (**Fig 4D** and **S4 Table**), which stimulates NF-κB signal transduction via activation of p65/RelA and trigger inflammatory responses [44, 45]. Previous study had demonstrated that IKBKE knockout attenuated inflammation and had a protective effective against diet-induced obesity [46]. We also noted that lariciresinol-4-β-D-glucopyranoside treated decreased the NF-κB-regulated genes encoding for the acute phase marker SAA1 [47] and inflammatory cytokine IL-6 were down-regulated (8.84 and 2.41-fold decrease, respectively) (**Fig 4D** and **S4 Table**), which reflects the systemic inflammatory response and key factor in symptom formation in influenza. These data are consistent with our previous finding that lariciresinol-4-β-D-glucopyranoside inhibited influenza virus-induced pro-inflammatory reaction via down-regulated the activation of NF-κB.

Moreover, TRIM25 is a key component of the RIG-I signaling pathway and subsequently initiate host antiviral immunity to RNA viruses infection [48], which was up-regulated 2.23-fold by lariciresinol-4-β-D-glucopyranoside treatment (**Fig 4D** and **S4 Table**). The vital role of TRIM25 in the IFN induction and RIG-I-dependent restriction of multiple RNA viruses has emerged from TRIM25-deficient study [48, 49]. The up-regulation of TRIM25 suggests that the transcript levels of the RIG-I signalling downstream genes type I/III interferons may also be elevated in infected cells with lariciresinol-4-β-D-glucopyranoside treatment. Accordingly, our results also clearly demonstrated that the expression of type III interferons (IFNL2 and IFNL3) were increased (1.93 and 1.90-fold increase, respectively) by lariciresinol-4-β-D-glucopyranoside treatment (**Fig 4D** and **S4 Table**). Interferons exerts their antiviral activity by inducing expression thousands of IFN-inducible antiviral effectors, such as MX1, OAS, IGS15, viperin and IFITM3 [50]. Interestingly, the expression of the IFN-inducible antiviral effector RSAD2 (radical S-adenosyl methionine domain containing 2, also known as viperin), belonging to the radical SAM superfamily that inhibits influenza virus release by disrupting lipid raft formation [51], was increased 3.17-fold as compared with that of viral-infected group (**Fig 4D** and **S4 Table**). But the expression of other the antiviral effector TRIM22, IFITM1, IFI27, BST2 (PDCA-1) and IFITM3 were not further up-regulated by lariciresinol-4-β-D-glucopyranoside treatment (**Fig 4D** and **S4 Table**). Increased expression of antiviral defence-related genes (TRIM25, IFNL2, IFNL3 and RSAD2) has provided some understanding into the mechanism of the antiviral effect of lariciresinol-4-β-D-glucopyranoside reported in our previous study, that’s lariciresinol-4-β-D-glucopyranoside treatment may enhance the host intrinsic antiviral immunity against viral infection.

There is growing evidencing that metabolism, such as lipid metabolism or amino acid (AA) metabolism, plays a potential role in viral infection [40, 52]. For instance, phospholipase D (PLD) catalyses of the hydrolysis phosphatidylcholine (PC), whereas it is reported that PLD can facilitate the entry of influenza virus [53]. Consistent with these, the present gene expression profiling showed some genes related to metabolism were altered influenza virus infection, such as LAT4H (leukotriene A4 hydrolase), IDO1(indoleamine-2,3-dioxygenase 1), PTGES (prostaglandin E synthase), ASS1 (argininosuccinate synthase 1) (**Fig 4B**, **Fig 4D** and **S4 Table**). PTGES expression was up-regulated upon influenza virus influenza and then contribute to PGE2 generation, which has been shown to increase viral replication via inhibition of IRF3-mediated type I IFN signaling pathway [38]. Infection with influenza virus has also been shown to increase the expression level of IDO1 in vitro and in vivo [54, 55]. Tryptophan (Trp) is an α-amino acid that can be metabolized by IDO1 to produce kynurenine (Kyn). Administration of a specific inhibitor of IDO led to elevation of influenza-specific CD8^+^ T-cells in influenza-infected mice [54]. IDO knockout mice were protected from lethal influenza challenge [56]. In our transcriptome data, gene expression of PTGES and IDO1 induced by influenza virus infection were down-regulated by lariciresinol-4-β-D-glucopyranoside treatment (**Fig 4B**, **Fig 4D**, **S4 Table** and **S8 Table**). These results suggest that lariciresinol-4-β-D-glucopyranoside modulated the host lipid metabolism and amino acid (AA) metabolism against influenza infection.

**Fig 6:**
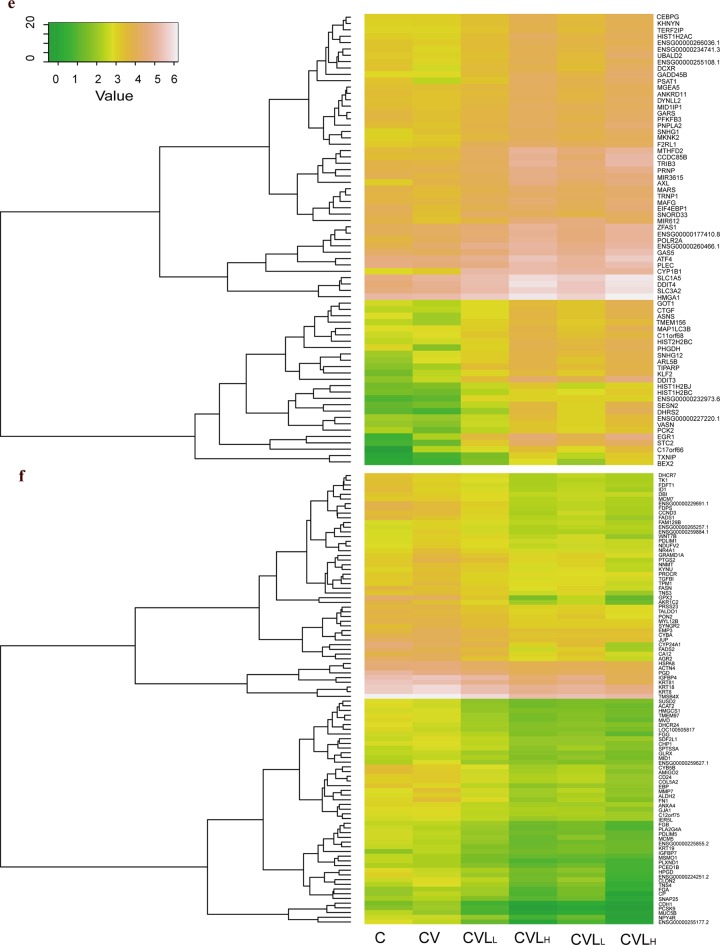
Heatmap depicting the emerging DEGs. **E)** Heatmap of 70 DEGs that was up-regulated solely by lariciresinol-4-β-D-glucopyranoside treatment (high expression: yellow; low expression: green). **F)** Heatmap of 96 DEGs that was down-regulated solely by lariciresinol-4-β-D-glucopyranoside treatment (high expression: yellow; low expression: green).

Functional enrichment analysis suggests that some DEGs regulated by lariciresinol-4-β-D-glucopyranoside treatment were involved in cell cycle and DNA repair (**Fig 4E** and **Fig 5C**). Indeed, it was showed that many of the DEGs related to cell cycle (CDKN1A, CCND3, GADD45a, GADD45b, MCM5, MCM7, ID1) and DNA damage (ATF3, ATF4, TIPARP, BBC3) were observed in the infected-cell with or without lariciresinol-4-β-D-glucopyranoside treatment (**Fig 4D**, **Fig 6E** and **Fig 6F**). Many viruses manipulate host cell division cycle to support their own replication [57–59]. Cell cycle analysis demonstrated that the induction of G0/G1 phase cell cycle arrest is associated with influenza virus efficient replication [9, 11]. Compared to cell cycle arrest at G2/M phase, cell cycle G0/G1 phase arrest is the most optimal condition for influenza virus protein synthesis [9]. Our results revealed that lariciresinol-4-β-D-glucopyranoside treatment increased expression of GADD45 (GADD45A, GADD45B) (**Fig 4D**, **Fig 6E**, **S4 Table** and **S7 Table**). In fact, many studies have found overexpression of GADD45, one of p53 target genes in response to DNA damage, causes G2/M cell cycle arrest in different human cell lines [60, 61]. Based upon the accumulated evidence and our findings, we have reasonable to speculate lariciresinol-4-β-D-glucopyranoside up-regulated the expression of GADD45 leading to cell cycle arrest at G2/M phase and decreased the synthesis of influenza virus proteins. Likewise, overexpression of ID1 inhibited the phosphorylation of p16^INK4a^ and RhoA [62]. It was found that inhibition of phosphorylation of p16^INK4a^ and RhoA by influenza virus NS1 protein were correlated with cell cycle G0/G1 phase arrest [9], thus establishing favorable conditions for progeny virus production. Although the expression of ID1 in the influenza virus-infected cells (CV) was comparable to levels in uninfected cells (C), lariciresinol-4-β-D-glucopyranoside treatment showed a prominent decrease in the expression of ID1 (**Fig 6F** and **S7 Table**). Thereby, we suppose that the down-regulation of ID1 by lariciresinol-4-β-D-glucopyranoside leads to increase the activity of p16^INK4a^ and RhoA, which would inhibit the cell cycle arrest at the G0/G1 cell phase for viral replication.

As identifying novel molecular targets involved in lariciresinol-4-β-D-glucopyranoside treatment is important, we observed the up-regulation of telomere end-packaging genes, such as HIST1H2AC, HIST1H2BC, HIST1H2BJ, MID1IP1 and TERF2IP (**Fig 6E** and **S8 Table**). However, the expression of these genes (HIST1H2AC, HIST1H2BC and MID1IP1) were only slightly down-regulated in influenza virus-infected cells (CV), but did not reach statistical significance (**S2 Fig**). Telomeres are essential for chromosome end-protection and genome stability. Dysregulation of telomeres has been implicated in many human diseases, including atherosclerosis, cancer, cardiovascular and infectious diaseaes [63–66]. Virus infection has been reported to alter host cell telomere maintenance and shorten the length of telomeres, which lead to DNA damage [66–68]. Vaccinia virus (VACV)- and cytomegalovirus (CMV)-specific CD4^+^ T cells have longer telomere lengths than IAV-specific CD4^+^ T cells [68]. A previous study reported that HeLa cells with influenza virus/B/Lee/40 infection significantly decreased the activity of telomerase on day 18 post-infection [69]. A recent study reveals that influenza virus-induced inflammation contribute to cellular oxidative DNA damage, which may play a role in the pathogenesis of influenza diseases [70]. Down-regulated expression of TERF2IP has been shown to trigger telomere dysfunction-induced DNA damage [71, 72], whereas lariciresinol-4-β-D-glucopyranoside treatment increased expression of TERF2IP. Although we did not observe prominent changes in the gene expression of the packaging of telomere ends by influenza A virus infection. We postulated that maybe the time-point of infection do not alter the expression of telomere-associated genes. These results suggests that lariciresinol-4-β-D-glucopyranoside may have beneficial effect in telomere protection and thus protect from influenza virus-induced telomere-related DNA damage.

## Conclusions

Our data provide insight into the transcriptome profile of host responses that are involved in the complex interplay of molecular pathways of virus-host interactions at the cellular level in lung epithelial A549 cells and a systematic understanding of the pharmacological mechanism of lariciresinol-4-β-D-glucopyranoside against viral infection.

## Supporting information

S1 Fig Enrichment analysis of 125 DEGs, which were down-regulated by influenza virus infection. Enrichment analysis showed that 125 DEGs which were down-regulated by influenza virus infection but not altered by lariciresinol-4-β-D-glucopyranoside treatment, enriched in the metabolism, gene expression and metabolism of proteins.
(TIF)

S2 Fig The DEGs located in the packaging of telomere ends pathway in lariciresinol-4-β-D-glucopyranoside treatments cells. The expression of these genes was altered at least fold change ≥ ± 1.5 and p-value < 0.05, but not in accordance with the criteria in virus-infected group (CV).
(TIF)

S1 Table List of influenza A virus-induced DEGs which was not altered by lariciresinol-4-β-D-glucopyranoside treatment. The expression of these genes was altered at least fold change ≥ ± 1.5 and p-value < 0.05.
(XLSX)

S2 Table Enriched GO categories and KEGG pathway of influenza A virus-induced DEGs, which were not altered by lariciresinol-4-β-D-glucopyranoside treatment. GO enrichment analysis showed that these DEGs induced by influenza virus infection were enriched in antiviral defence, type I interferon signaling pathway and innate immune response. KEGG pathway enrichment analysis showed that these DEGs induced by influenza virus infection were enriched in pathways such as TNF signaling pathway, NOD-like receptor signaling pathway and chemokine signaling pathway.
(XLSX)

S3 Table The enriched KEGG pathway of 125 DEGs, which were down-regulated by influenza virus infection. KEGG pathway enrichment analysis showed that 125 DEGs, which were down-regulated by influenza virus infection but not altered by lariciresinol-4-β-D-glucopyranoside treatment, enriched in 28 pathways such as L13a-mediated translational silencing of Ceruloplasmin expression, eukaryotic Translation Initiation and synthesis of bile acids and bile salts via 27-hydroxycholesterol.
(XLSX)

S4 Table List of influenza A virus-induced DEGs, which were directly regulated by lariciresinol-4-β-D-glucopyranoside treatment. The expression of these genes was altered at least fold change ≥ ± 1.5 and p-value < 0.05.
(XLSX)

S5 Table Enriched GO categories and KEGG pathway of influenza A virus-induced DEGs, which were directly regulated by lariciresinol-4-β-D-glucopyranoside treatment. GO enrichment analysis showed these DEGs directly regulated by lariciresinol-4-β-D-glucopyranoside were enriched in defence response to virus, immune response and antiviral defence. KEGG pathway enrichment analysis showed that these DEGs were enriched in pathways such as chemokine signaling pathway, cytokine-cytokine receptor interaction and T cell receptor signaling pathway.
(XLSX)

S6 Table The enriched KEGG pathway of 62 DEGs, which were down-regulated by lariciresinol-4-β-D-glucopyranoside treatment. KEGG pathway enrichment analysis showed that 62 DEGs down-regulated by lariciresinol-4-β-D-glucopyranoside treatment were enriched in pathways such as interleukin-7 signaling, prolactin receptor signaling and signaling by FGFR1 fusion mutants.
(XLSX)

S7 Table List of emerging DEGs altered solely by lariciresinol-4-β-D-glucopyranoside treatment. Genes expression altered fold change ≥ ± 1.5 and p-value < 0.05 were considered as emerging DEGs, but not in accordance with the criteria in virus-infected group (CV).
(XLSX)

S8 Table Enrichment analysis of biological process GO terms of emerging DEGs by lariciresinol-4-β-D-glucopyranoside treatment. Genes expression that up-regulated or down-regulated at least fold change ≥ ± 1.5 and p-value < 0.05 were considered as emerging DEGs, but not in accordance with the criteria in virus-infected group (CV).
(XLSX)

S9 Table The enriched KEGG pathway of 96 emerging DEGs, which were down-regulated solely by lariciresinol-4-β-D-glucopyranoside treatment. These emerging DEGs (Genes expression were down-regulated by at least 1.5-fold change and p-value < 0.05 were considered as emerging DEGs, but the expression of these DEGs not in accordance with the criteria in virus-infected group (CV)) were enriched in 46 pathways such as activation of gene expression by SREBF, cholesterol biosynthesis,linoleic acid (LA) metabolism and MAP2K and MAPK activation.
(XLSX)
