# Supplementary figures and images for: Transcriptome profiling of influenza A virus-infected lung epithelial (A549) cells with lariciresinol-4-β-D-glucopyranoside treatment
Source: PLoS One. 2017 Mar 8;12(3):e0173058. doi: 10.1371/journal.pone.0173058 (PMC5342222; doi:10.1371/journal.pone.0173058)

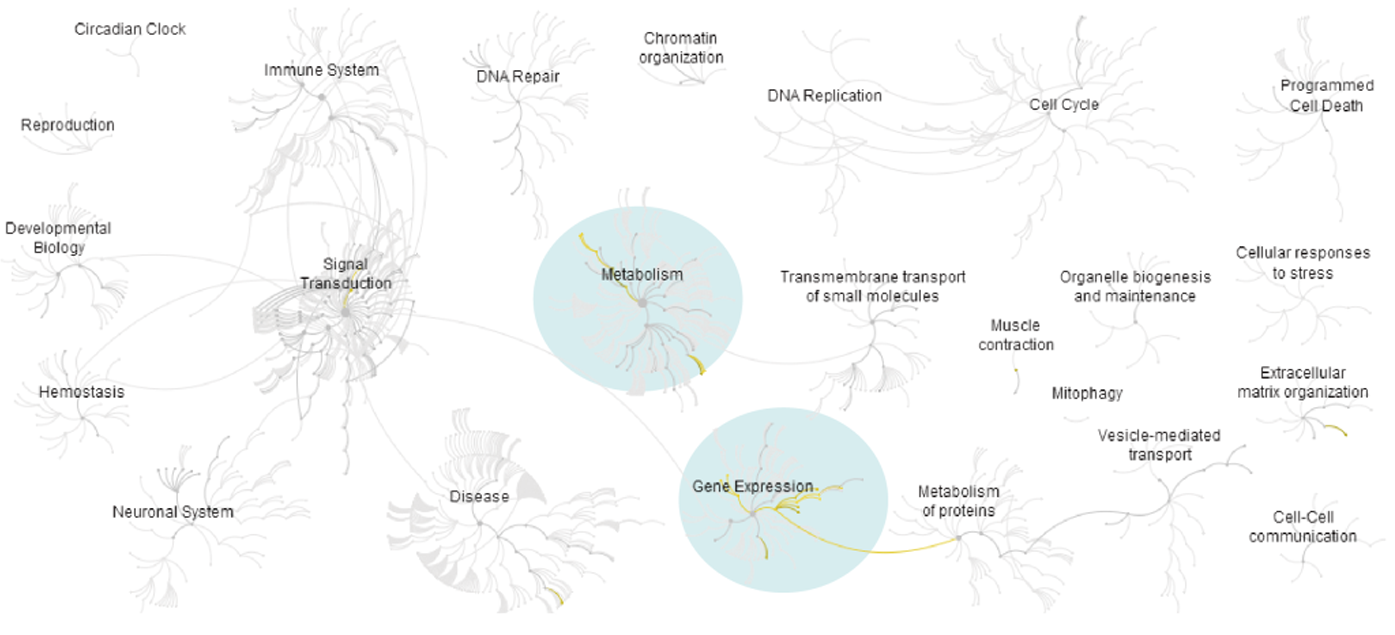

Supplement: S1 Fig — Enrichment analysis showed that 125 DEGs which were down-regulated by influenza virus infection but not altered by lariciresinol-4-β-D-glucopyranoside treatment, enriched in the metabolism, gene expression and metabolism of proteins. (TIF) [file pone.0173058.s001.tif]

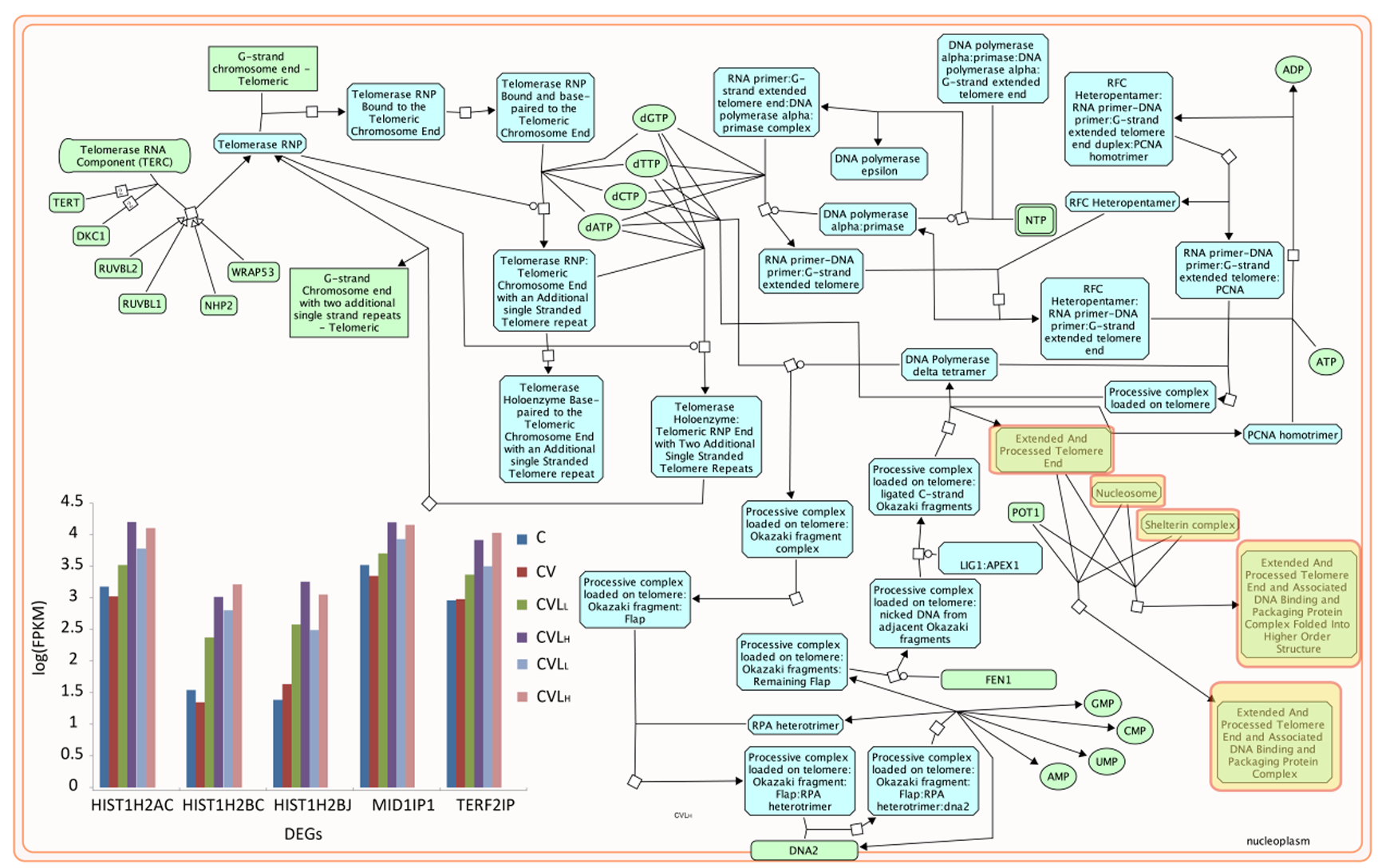

Supplement: S2 Fig — The expression of these genes was altered at least fold change ≥ ± 1.5 and p-value < 0.05, but not in accordance with the criteria in virus-infected group (CV). (TIF) [file pone.0173058.s002.tif]
